# Ethyl anthracene-9-carboxyl­ate

**DOI:** 10.1107/S1600536808017819

**Published:** 2008-06-19

**Authors:** Edwin Weber, Wilhelm Seichter, Conrad Fischer, L. M. S. Franziska Bendrath, Bakhtiyar T. Ibragimov

**Affiliations:** a Institut für Organische Chemie, TU Bergakademie Freiberg, Leipziger Strasse 29, D-09596 Freiberg/Sachsen, Germany; b Institute of Bioorganic Chemistry, Academy of Sciences of Uzbekistan, H Abdullaev 83, Tashkent 100125, Uzbekistan

## Abstract

In the title compound, C_17_H_14_O_2_, the COO group and the anthracene fragment form a dihedral angle of 76.00 (19)°. The torsion angle around the O—C*sp* ^3^ bond of the ester group is 108.52 (18)°. The crystal structure is stabilized by C—H⋯O inter­actions and edge-to-face arene inter­actions with C—H⋯(ring centroid) distances in the range 2.75–2.84 Å.

## Related literature

For related crystal structures, see: Bart & Schmidt (1971 ▶); Heller & Schmidt (1971 ▶); Sweeting *et al.* (1997 ▶). For the preparation of the title compound, see: Larsen & Harpp (1980 ▶).
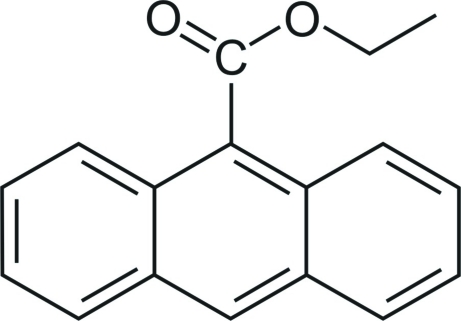


## Experimental

### 

#### Crystal data

C_17_H_14_O_2_
*M* *_r_* = 250.28
Orthorhombic, 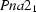

*a* = 8.5431 (6) Å
*b* = 10.2137 (7) Å
*c* = 14.5426 (11) Å
*V* = 1268.94 (16) Å^3^
*Z* = 4
Mo *K*α radiation
μ = 0.09 mm^−1^
*T* = 153 (2) K
0.25 × 0.25 × 0.20 mm

#### Data collection

Bruker Kappa APEXII CCD diffractometer
Absorption correction: none
15373 measured reflections
2020 independent reflections
1600 reflections with *I* > 2σ(*I*)
*R*_int_ = 0.047

#### Refinement

*R*[*F* ^2^ > 2σ(*F* ^2^)] = 0.035
*wR*(*F* ^2^) = 0.085
*S* = 1.04
2020 reflections
174 parameters
1 restraint
H-atom parameters constrained
Δρ_max_ = 0.22 e Å^−3^
Δρ_min_ = −0.17 e Å^−3^

### 

Data collection: *APEX2* (Bruker, 2004 ▶); cell refinement: *SAINT* (Bruker, 2004 ▶); data reduction: *SAINT*; program(s) used to solve structure: *SHELXS97* (Sheldrick, 2008 ▶); program(s) used to refine structure: *SHELXL97* (Sheldrick, 2008 ▶); molecular graphics: *ORTEP-3* (Farrugia, 1997 ▶); software used to prepare material for publication: *SHELXTL* (Sheldrick, 2008 ▶).

## Supplementary Material

Crystal structure: contains datablocks global, I. DOI: 10.1107/S1600536808017819/gk2145sup1.cif

Structure factors: contains datablocks I. DOI: 10.1107/S1600536808017819/gk2145Isup2.hkl

Additional supplementary materials:  crystallographic information; 3D view; checkCIF report

## Figures and Tables

**Table 1:** Hydrogen-bond geometry (Å, °)

*D*—H⋯*A*	*D*—H	H⋯*A*	*D*⋯*A*	*D*—H⋯*A*
C5—H5⋯O1^i^	0.93	2.53	3.302 (2)	140

Symmetry code: (i) 
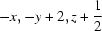
.

**Fig. 1.**
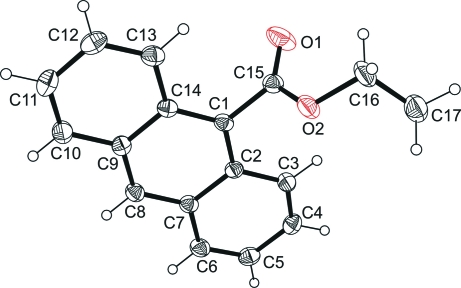
Molecular structure of the title compound with atomic labels and 50% probability displacement ellipsoids for non H-atoms.

**Fig. 2.**
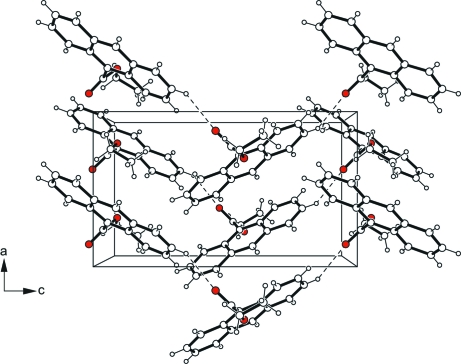
Crystal packing of the title compound viewed along the b axis.

## supplementary crystallographic information

### Comment

9-Anthracenecarboxylic acid esters are of current interest in materials science (Sweeting *et al.*, 1997). The conformational features of the title compound (Fig. 1) resemble those found in the crystal structure of the analogous methyl 9-anthracenecarboxylate (Bart & Schmidt, 1971). A comparative examination of the crystal structures, however, reveals that a slight modification of the molecular structure has a fundamental influence on the molecular packing mode. According to the presence of a twofold screw axis, helical hydrogen bonded strands (Table 1, Fig. 2) running along the *c* axis are the basic supramolecular entities of the present crystal structure. Furthermore, the anthracene units of neighbouring strands are arranged in "edge-to-face" herringbone fashion with the closest intermolecular distance of 2.86 Å.

### Experimental

9-Anthracenecarbonyl chloride (300 mg) in CH_2_Cl_2_ (45 ml) was reacted with ethanol (10 ml) and pyridine (2 ml). The resulting solution was heated under reflux for 11 h, then cooled to room temperature and subsequently extracted three times with 2 N aqueous HCl and water (50 ml, each), and finally two times with water (100 ml). After addition of CH_2_Cl_2_ (200 ml) the organic layer was dried over CaCl_2_ and the solvent removed under reduced pressure. Recrystallization of the white powder from acetone yielded colourless crystals suitable for X-ray diffraction analysis. (82%, m.p. 381–382 K). Anal. Calcd. for C_17_H_14_O_2_: C 81.58; H 5.64; Found: C 81.42; H 5.90%.

### Refinement

In absence of significant anomalous scattering effects, Friedel pairs were merged prior to refinement. All hydrogen atoms were positioned geometrically and refined using the riding model with d(C—H) = 0.93 Å, *U*_iso_ = 1.2Ueq(C) for aromatic, 0.96 Å, *U*_iso_ = 1.5Ueq(C) for CH_3_ and 0.97 Å, *U*_iso_ = 1.2Ueq(C) for CH_2_ H atoms.

### Crystal data

**Table:**

C_17_H_14_O_2_	*F*_000_ = 528
*M**_r_* = 250.28	*D*_x_ = 1.310 Mg m^−^^3^
Orthorhombic, *P**n**a*2_1_	Mo *K*α radiation λ = 0.71073 Å
Hall symbol: P 2c -2n	Cell parameters from 4881 reflections
*a* = 8.5431 (6) Å	θ = 2.4–30.5º
*b* = 10.2137 (7) Å	µ = 0.09 mm^−^^1^
*c* = 14.5426 (11) Å	*T* = 153 (2) K
*V* = 1268.94 (16) Å^3^	Irregular, colourless
*Z* = 4	0.25 × 0.25 × 0.20 mm

### Data collection

**Table:**

Bruker Kappa APEXII CCD diffractometer	1600 reflections with *I* > 2σ(*I*)
Radiation source: fine-focus sealed tube	*R*_int_ = 0.047
Monochromator: graphite	θ_max_ = 30.6º
*T* = 153(2) K	θ_min_ = 2.4º
φ and ω scans	*h* = −11→12
Absorption correction: none	*k* = −12→14
15373 measured reflections	*l* = −20→13
2020 independent reflections	

### Refinement

**Table:**

Refinement on *F*^2^	Secondary atom site location: difference Fourier map
Least-squares matrix: full	Hydrogen site location: inferred from neighbouring sites
*R*[*F*^2^ > 2σ(*F*^2^)] = 0.035	H-atom parameters constrained
*wR*(*F*^2^) = 0.085	*w* = 1/[σ^2^(*F*_o_^2^) + (0.0459*P*)^2^] where *P* = (*F*_o_^2^ + 2*F*_c_^2^)/3
*S* = 1.04	(Δ/σ)_max_ < 0.001
2020 reflections	Δρ_max_ = 0.22 e Å^−^^3^
174 parameters	Δρ_min_ = −0.17 e Å^−^^3^
1 restraint	Extinction correction: none
Primary atom site location: structure-invariant direct methods	

### Special details

**Table:**

Experimental. ^1^H-NMR (400 MHz, CDCl_3_, δ, p.p.m.): 1.53 (m, CH_3_); 4.68 (q, ^3^J=7.2 Hz, OCH_2_, 2H); 7.45 (m, H_2_, H_3_, H_6_, H_7_, 4H); 8.03 (t, H_1_, H_4_, H_5_, H_8_, 4H); 8.54 (t, H_10_, 1H). ^13^C-NMR (100 MHz, CDCl_3_, δ, p.p.m.): 13.70 (CH_3_), 61.70 (OCH_2_), 125.17 (C_1_, C_8_), 125.86 (C_3_, C_6_), 127.25 (C_2_, C_7_); 128.37 (C_9_, C_4a_, C_10_*_a_*); 128.89 (C_4_, C_5_); 129.23 (C_10_); 131.35 (C_8a_, C_9a_); 169.10 (CŌ). IR (KBr, cm^-1^): 3079 (w), 3053 (w)(C–H_ar_); 2981 (*m*), 2929, 2904, 2867 (C–H); 1952; 1802; 1715 (C?O); 1626; 1564; 1522; 1467; 1455; 1420; 1388; 1372; 1352; 1321; 1288; 1264; 1238; 1216; 1171; 1151; 1119; 1099; 1025; 974; 957; 935; 897; 866; 846; 810; 740; 671; 633; 607; 560; 529; 452. GC—MS *m/z* 250 (100, *M*^+^), 235, 222, 205, 177, 151, 139, 126, 102, 88, 75, 51.
Geometry. All e.s.d.'s (except the e.s.d. in the dihedral angle between two l.s. planes) are estimated using the full covariance matrix. The cell e.s.d.'s are taken into account individually in the estimation of e.s.d.'s in distances, angles and torsion angles; correlations between e.s.d.'s in cell parameters are only used when they are defined by crystal symmetry. An approximate (isotropic) treatment of cell e.s.d.'s is used for estimating e.s.d.'s involving l.s. planes.
Refinement. Refinement of *F*^2^ against ALL reflections. The weighted *R*-factor *wR* and goodness of fit *S* are based on *F*^2^, conventional *R*-factors *R* are based on *F*, with *F* set to zero for negative *F*^2^. The threshold expression of *F*^2^ > σ(*F*^2^) is used only for calculating *R*-factors(gt) *etc*. and is not relevant to the choice of reflections for refinement. *R*-factors based on *F*^2^ are statistically about twice as large as those based on *F*, and *R*- factors based on ALL data will be even larger.

### Fractional atomic coordinates and isotropic or equivalent isotropic displacement parameters (Å^2^)

**Table:**

	*x*	*y*	*z*	*U*_iso_*/*U*_eq_	
O1	0.13453 (17)	0.82389 (13)	−0.03306 (11)	0.0430 (4)	
O2	0.30770 (15)	0.75339 (12)	0.07149 (10)	0.0316 (3)	
C1	0.28393 (19)	0.97928 (15)	0.05261 (11)	0.0195 (3)	
C2	0.23153 (19)	1.03155 (15)	0.13680 (11)	0.0198 (3)	
C3	0.1270 (2)	0.96373 (17)	0.19647 (11)	0.0233 (4)	
H3	0.0919	0.8805	0.1806	0.028*	
C4	0.0777 (2)	1.01972 (17)	0.27676 (12)	0.0264 (4)	
H4	0.0082	0.9747	0.3145	0.032*	
C5	0.1310 (2)	1.14542 (18)	0.30335 (12)	0.0269 (4)	
H5	0.0973	1.1818	0.3585	0.032*	
C6	0.2313 (2)	1.21299 (17)	0.24865 (12)	0.0249 (4)	
H6	0.2662	1.2951	0.2672	0.030*	
C7	0.2840 (2)	1.16012 (15)	0.16313 (11)	0.0206 (3)	
C8	0.3840 (2)	1.22952 (16)	0.10477 (12)	0.0222 (3)	
H8	0.4185	1.3121	0.1226	0.027*	
C9	0.4334 (2)	1.17882 (15)	0.02076 (11)	0.0210 (3)	
C10	0.5365 (2)	1.24932 (16)	−0.03850 (12)	0.0269 (4)	
H10	0.5701	1.3325	−0.0216	0.032*	
C11	0.5865 (2)	1.19688 (18)	−0.11930 (14)	0.0307 (4)	
H11	0.6544	1.2441	−0.1567	0.037*	
C12	0.5355 (2)	1.07060 (19)	−0.14688 (12)	0.0294 (4)	
H12	0.5700	1.0358	−0.2024	0.035*	
C13	0.4366 (2)	1.00008 (16)	−0.09291 (11)	0.0256 (4)	
H13	0.4040	0.9176	−0.1122	0.031*	
C14	0.38172 (19)	1.05069 (16)	−0.00681 (11)	0.0201 (3)	
C15	0.2313 (2)	0.84551 (16)	0.02446 (12)	0.0224 (3)	
C16	0.2630 (2)	0.61657 (16)	0.05647 (15)	0.0336 (4)	
H16A	0.3556	0.5640	0.0454	0.040*	
H16B	0.1954	0.6100	0.0031	0.040*	
C17	0.1792 (2)	0.56759 (19)	0.13964 (15)	0.0366 (5)	
H17A	0.2438	0.5802	0.1929	0.055*	
H17B	0.1569	0.4760	0.1324	0.055*	
H17C	0.0830	0.6150	0.1471	0.055*	

### Atomic displacement parameters (Å^2^)

**Table:**

	*U*^11^	*U*^22^	*U*^33^	*U*^12^	*U*^13^	*U*^23^
O1	0.0469 (9)	0.0323 (8)	0.0498 (9)	−0.0006 (6)	−0.0262 (8)	−0.0080 (6)
O2	0.0389 (8)	0.0185 (5)	0.0374 (7)	−0.0030 (5)	−0.0137 (6)	−0.0007 (5)
C1	0.0205 (8)	0.0176 (7)	0.0204 (7)	0.0016 (6)	−0.0040 (6)	−0.0016 (6)
C2	0.0201 (8)	0.0202 (7)	0.0192 (7)	0.0007 (6)	−0.0040 (6)	0.0016 (6)
C3	0.0239 (9)	0.0218 (8)	0.0243 (9)	−0.0021 (6)	−0.0015 (7)	0.0004 (6)
C4	0.0260 (9)	0.0292 (9)	0.0239 (8)	−0.0010 (7)	0.0038 (7)	0.0037 (7)
C5	0.0303 (10)	0.0298 (9)	0.0204 (8)	0.0032 (7)	0.0022 (7)	−0.0041 (7)
C6	0.0293 (10)	0.0210 (8)	0.0245 (8)	0.0005 (7)	−0.0007 (8)	−0.0047 (7)
C7	0.0220 (9)	0.0190 (7)	0.0208 (7)	0.0012 (6)	−0.0019 (6)	−0.0014 (6)
C8	0.0241 (9)	0.0190 (8)	0.0235 (8)	−0.0008 (6)	−0.0015 (7)	−0.0016 (6)
C9	0.0203 (8)	0.0203 (7)	0.0225 (7)	0.0015 (6)	−0.0011 (7)	0.0012 (6)
C10	0.0262 (10)	0.0257 (8)	0.0289 (9)	−0.0027 (7)	−0.0001 (7)	0.0017 (7)
C11	0.0293 (10)	0.0337 (10)	0.0292 (9)	−0.0003 (8)	0.0066 (8)	0.0068 (8)
C12	0.0298 (10)	0.0361 (10)	0.0222 (8)	0.0067 (8)	0.0039 (7)	−0.0006 (7)
C13	0.0286 (10)	0.0246 (8)	0.0235 (8)	0.0021 (7)	−0.0007 (7)	−0.0039 (6)
C14	0.0198 (8)	0.0206 (7)	0.0200 (8)	0.0025 (6)	−0.0021 (6)	−0.0014 (6)
C15	0.0232 (9)	0.0235 (7)	0.0205 (7)	−0.0007 (6)	0.0009 (7)	−0.0026 (7)
C16	0.0422 (11)	0.0181 (8)	0.0407 (10)	−0.0045 (7)	−0.0063 (9)	−0.0034 (7)
C17	0.0353 (11)	0.0302 (10)	0.0443 (11)	−0.0068 (8)	−0.0071 (9)	0.0026 (9)

### Geometric parameters (Å, °)

**Table:**

O1—C15	1.197 (2)	C8—H8	0.9300
O2—C15	1.334 (2)	C9—C10	1.427 (2)
O2—C16	1.465 (2)	C9—C14	1.438 (2)
C1—C14	1.406 (2)	C10—C11	1.360 (3)
C1—C2	1.409 (2)	C10—H10	0.9300
C1—C15	1.495 (2)	C11—C12	1.419 (3)
C2—C3	1.425 (2)	C11—H11	0.9300
C2—C7	1.439 (2)	C12—C13	1.360 (3)
C3—C4	1.367 (2)	C12—H12	0.9300
C3—H3	0.9300	C13—C14	1.433 (2)
C4—C5	1.416 (3)	C13—H13	0.9300
C4—H4	0.9300	C16—C17	1.492 (3)
C5—C6	1.357 (3)	C16—H16A	0.9700
C5—H5	0.9300	C16—H16B	0.9700
C6—C7	1.429 (2)	C17—H17A	0.9600
C6—H6	0.9300	C17—H17B	0.9600
C7—C8	1.397 (2)	C17—H17C	0.9600
C8—C9	1.393 (2)		
			
C15—O2—C16	117.96 (15)	C11—C10—H10	119.4
C14—C1—C2	121.76 (14)	C9—C10—H10	119.4
C14—C1—C15	118.97 (15)	C10—C11—C12	120.39 (17)
C2—C1—C15	119.25 (15)	C10—C11—H11	119.8
C1—C2—C3	122.97 (14)	C12—C11—H11	119.8
C1—C2—C7	118.56 (14)	C13—C12—C11	120.60 (17)
C3—C2—C7	118.46 (14)	C13—C12—H12	119.7
C4—C3—C2	120.66 (16)	C11—C12—H12	119.7
C4—C3—H3	119.7	C12—C13—C14	121.09 (16)
C2—C3—H3	119.7	C12—C13—H13	119.5
C3—C4—C5	120.90 (17)	C14—C13—H13	119.5
C3—C4—H4	119.6	C1—C14—C13	122.97 (15)
C5—C4—H4	119.6	C1—C14—C9	118.88 (14)
C6—C5—C4	120.27 (16)	C13—C14—C9	118.12 (15)
C6—C5—H5	119.9	O1—C15—O2	124.50 (15)
C4—C5—H5	119.9	O1—C15—C1	124.58 (15)
C5—C6—C7	121.11 (16)	O2—C15—C1	110.92 (14)
C5—C6—H6	119.4	O2—C16—C17	108.92 (16)
C7—C6—H6	119.4	O2—C16—H16A	109.9
C8—C7—C6	121.98 (15)	C17—C16—H16A	109.9
C8—C7—C2	119.44 (14)	O2—C16—H16B	109.9
C6—C7—C2	118.58 (15)	C17—C16—H16B	109.9
C9—C8—C7	121.98 (15)	H16A—C16—H16B	108.3
C9—C8—H8	119.0	C16—C17—H17A	109.5
C7—C8—H8	119.0	C16—C17—H17B	109.5
C8—C9—C10	121.96 (15)	H17A—C17—H17B	109.5
C8—C9—C14	119.34 (15)	C16—C17—H17C	109.5
C10—C9—C14	118.69 (15)	H17A—C17—H17C	109.5
C11—C10—C9	121.11 (16)	H17B—C17—H17C	109.5
			
C14—C1—C2—C3	177.19 (15)	C9—C10—C11—C12	0.6 (3)
C15—C1—C2—C3	−1.3 (2)	C10—C11—C12—C13	−0.2 (3)
C14—C1—C2—C7	−1.8 (2)	C11—C12—C13—C14	−0.3 (3)
C15—C1—C2—C7	179.65 (14)	C2—C1—C14—C13	−179.90 (15)
C1—C2—C3—C4	−178.95 (16)	C15—C1—C14—C13	−1.4 (2)
C7—C2—C3—C4	0.1 (2)	C2—C1—C14—C9	2.0 (2)
C2—C3—C4—C5	−1.0 (3)	C15—C1—C14—C9	−179.43 (15)
C3—C4—C5—C6	0.7 (3)	C12—C13—C14—C1	−177.67 (17)
C4—C5—C6—C7	0.6 (3)	C12—C13—C14—C9	0.4 (3)
C5—C6—C7—C8	178.35 (17)	C8—C9—C14—C1	−0.8 (2)
C5—C6—C7—C2	−1.5 (3)	C10—C9—C14—C1	178.09 (15)
C1—C2—C7—C8	0.3 (2)	C8—C9—C14—C13	−178.94 (15)
C3—C2—C7—C8	−178.70 (16)	C10—C9—C14—C13	−0.1 (2)
C1—C2—C7—C6	−179.79 (16)	C16—O2—C15—O1	−4.4 (3)
C3—C2—C7—C6	1.2 (2)	C16—O2—C15—C1	176.17 (15)
C6—C7—C8—C9	−178.99 (16)	C14—C1—C15—O1	−74.0 (2)
C2—C7—C8—C9	0.9 (3)	C2—C1—C15—O1	104.6 (2)
C7—C8—C9—C10	−179.49 (16)	C14—C1—C15—O2	105.43 (17)
C7—C8—C9—C14	−0.7 (3)	C2—C1—C15—O2	−76.00 (19)
C8—C9—C10—C11	178.42 (18)	C15—O2—C16—C17	−108.52 (18)
C14—C9—C10—C11	−0.4 (3)		

### Hydrogen-bond geometry (Å, °)

**Table:**

*D*—H···*A*	*D*—H	H···*A*	*D*···*A*	*D*—H···*A*
C5—H5···O1^i^	0.93	2.53	3.302 (2)	140

Symmetry codes: (i) −*x*, −*y*+2, *z*+1/2.
